# Supplementary material for: How common are high-risk coronavirus contacts? A video-observational analysis of outdoor public place behavior during the COVID-19 pandemic
Source: PLoS One. 2022 Mar 17;17(3):e0265680. doi: 10.1371/journal.pone.0265680 (PMC8929547; doi:10.1371/journal.pone.0265680)
Supplement: Attachment — Submitted filename: Response to Reviewers (2).docx [file pone.0265680.s002.docx]

***** Response to reviews of ‘How common are high-risk COVID-19 contacts?’******

***Editors feedback***

1. **Please ensure that your manuscript meets PLOS ONE's style requirements, including those for file naming. The PLOS ONE style templates can be found at** [**https://journals.plos.org/plosone/s/file?id=wjVg/PLOSOne_formatting_sample_main_body.pdf**](https://journals.plos.org/plosone/s/file?id=wjVg/PLOSOne_formatting_sample_main_body.pdf) **and** [**https://journals.plos.org/plosone/s/file?id=ba62/PLOSOne_formatting_sample_title_authors_affiliations.pdf**](https://journals.plos.org/plosone/s/file?id=ba62/PLOSOne_formatting_sample_title_authors_affiliations.pdf)

***REPLY: Validated.

2. **In your Data Availability statement, you have not specified where the minimal data set underlying the results described in your manuscript can be found. PLOS defines a study's minimal data set as the underlying data used to reach the conclusions drawn in the manuscript and any additional data required to replicate the reported study findings in their entirety. All PLOS journals require that the minimal data set be made fully available. For more information about our data policy, please see** [**http://journals.plos.org/plosone/s/data-availability**](http://journals.plos.org/plosone/s/data-availability)**.**

**Upon re-submitting your revised manuscript, please upload your study’s minimal underlying data set as either Supporting Information files or to a stable, public repository and include the relevant URLs, DOIs, or accession numbers within your revised cover letter. For a list of acceptable repositories, please see** [**http://journals.plos.org/plosone/s/data-availability#loc-recommended-repositories**](http://journals.plos.org/plosone/s/data-availability#loc-recommended-repositories)**. Any potentially identifying patient information must be fully anonymized.**

**Important: If there are ethical or legal restrictions to sharing your data publicly, please explain these restrictions in detail. Please see our guidelines for more information on what we consider unacceptable restrictions to publicly sharing data: http://journals.plos.org/plosone/s/data-availability#loc-unacceptable-data-access-restrictions. Note that it is not acceptable for the authors to be the sole named individuals responsible for ensuring data access.**

***REPLY: Replication data and materials are available at https://osf.io/7ek9d/.

3. **We note that you have indicated that data from this study are available upon request. PLOS only allows data to be available upon request if there are legal or ethical restrictions on sharing data publicly. For more information on unacceptable data access restrictions, please see http://journals.plos.org/plosone/s/data-availability#loc-unacceptable-data-access-restrictions.**

**In your revised cover letter, please address the following prompts:**

**a) If there are ethical or legal restrictions on sharing a de-identified data set, please explain them in detail (e.g., data contain potentially sensitive information, data are owned by a third-party organization, etc.) and who has imposed them (e.g., an ethics committee). Please also provide contact information for a data access committee, ethics committee, or other institutional body to which data requests may be sent.**

**b) If there are no restrictions, please upload the minimal anonymized data set necessary to replicate your study findings as either Supporting Information files or to a stable, public repository and provide us with the relevant URLs, DOIs, or accession numbers. For a list of acceptable repositories, please see** [**http://journals.plos.org/plosone/s/data-availability#loc-recommended-repositories**](http://journals.plos.org/plosone/s/data-availability#loc-recommended-repositories)**.**

***REPLY: We would like to stress that all data scripts, transcripts, and materials are publicly available at <https://osf.io/7ek9d/>. Only the raw data, consisting of video recordings of individuals moving through public space, is available upon request. Ethical and legal restrictions for sharing this data are mentioned in the revised Data Availability statement.

***Reviewer #1***

4. **Authors have performed this study at outdoor public places in Amsterdam, it would be interesting to analyze other available data from other geographical location in the Netherlands.**

***REPLY: We agree that this would be interesting and relevant. However, in the current study, we only have access to CCTV camera footage from Amsterdam. In fact, we were only able to collect this data because of our long-term collaboration with the Amsterdam Police Department—there exists no centralized, nationwide archive of CCTV data. In the revised paper (p. 9), we have further flagged this geographical issue as a study limitation.

5. **Authors should provide their data graphical representable format.**

***REPLY: We agree that this would add clarity to the argument and have thus summarized the data patterns in Figure 1 (p. 5) and Figure 2 (p. 6).

6. **Authors should discuss their study by putting/citing some current research.**

***REPLY: We now cite and discuss other recent studies relating to the topic at hand, both in the introduction (p. 3) and the discussion section (p. 8-9).

7. **At least one supplementary Figure as illustration may be afforded as to highlight the summary or prospect of this study.**

***REPLY: In the exercise of trying to visualize our main results, we decided to create two figures. Figure 1 (p. 5) is a descriptive figure which summarizes the main findings with regard to the frequency of high-risk contacts, and Figure 2 (p. 6) is modelling based and details the difference between low and high risk distancing contacts. We have revised the Results section as part of the inclusion of these figures.

8. **What was the infection rate in Netherlands when this study was conducted and what might be the proposed reason for that?**

***REPLY: In the revised paper (p. 4), we now specify that the Netherlands case largely followed the European pattern, with fluctuating infection rates across the data collections period lasting one full year.

***Reviewer #2:***

9. **The authors thus conclude that their results alleviate the public health concern regarding coronavirus transmission in outdoor public places.**

**This conclusion may mislead. From the several waves of SARS-CoV-2 virus infection outbreak in the past two years in almost all major countries in the world, we can see that although the rate of outdoor person-to-person viral transmission is much lower than that of the indoor transmission, outdoor transmission is still a considerable form of transmission. The authors’ result rather shows that the WHO definition of high-risk contact is inadequate: long distance (greater than one meter) and short time (less than 15 minutes) can still constitute high risk of infection. This is because, the SARS-CoV-2 virus can be transmitted in airborne way [1-3].**

**The highly contagious nature of the SARS-CoV-2 viral infection shows that although facial mask may slow down the transmission of the virus, in long run, infection is inevitable, and we have to live with this SARS-CoV-2 virus. Epidemiologic data show that most of the COVID-19 cases are asymptomatic and mild [4] and the SARS-CoV-2 virus is self-limiting [5]. The severe cases of COVID-19 is dominantly caused by the immunopathology, i.e., the overreaction of the host immune system and the cytokine storm [6,7] instead of viral infection. So the public health authorities may focus more on the curing of the noncommunicable diseases like morbid obesity, type 2 diabetes mellitus and other metabolic syndromes than the concern regarding the coronavirus infection and transmission.**

***REPLY: We agree that the implications of our results could be nuanced along the suggested lines. The revised paper (p. 8-9) now unpacks these potential implications and considerations in a paragraph.

***Reviewer #3:***

10. **The English may be polished. There are grammatical errors and spelling mistakes in the manuscript.**

***REPLY: We have copy-edited the manuscript.

11. **The link provided in the manuscript in line 100 is not working. Find a way to make the data available to readers.**

***REPLY: We thank reviewer #3 for flagging this; the link is now updated.

12. **The cameras were located in busy settings such as shopping streets, public transportation. Will footage from pubs, grocery shops, coffee shops change the results?. Why did authors not consider these settings for their study as these sites are closer and human interaction is more?.**

***REPLY. We agree that this would be interesting, but the CCTV data systems we have obtained access to are only installed in outdoor public places. However, in the revised paper (p. 9), we now flag this as a study limitation. Further, in the discussion (p. 9), we now also consider the current result with respect to interaction in indoor vis-à-vis outdoor settings.

13. **The footage is from 1 p.m and 1.30 p.m; for half an hour in the afternoon. During this time, the rush in public transport is minor. Why did the authors not consider another time for their study?. Will footage time like office hours and school hours affect the results?**

***REPLY: We thank reviewer #3 for this comment, we now add a comment on why this period was selected, and furthermore, we acknowledge this as a study limitation that may have influenced the current results (p. 9).

14. **Please mention the limitation of the study?**

***REPLY: We have further detailed the study limitations (presented in the last paragraphs of the paper (p. 9).

15. **The authors may additionally provide one Figure as a summary in results section.**

***REPLY: In the exercise of trying to visualize our main results, we decided to create 2 figures. Figure 1 (p. 5) is a descriptive figure which summarizes the main findings with regard to the frequency of high-risk contacts, and Figure 2 (p. 6) is modelling based and details the difference between low and high risk distancing contacts. We have revised the Results section as part of the inclusion of these figures.

16. **The author may elaborate on the results of observational video analysis in the discussion.**

***REPLY: Thanks to this comment, the revised paper now elaborates/discusses the results of the analysis in more detail (p. 8). Relatedly, we have also expanded the introduction with citations and considerations of other research so as to clarify the contribution of the paper (p. 2-3).

17. **The authors discuss similar studies in the introduction and few information on social distancing, nature biomolecules for their treatment, and importance of health (few references i.e. doi: 10.1007/s12088-020-00908-0; doi: 10.1007/s12088-020-00893-4).**

***REPLY: Throughout the paper revision, we have considered additional relevant citations and have included several. We hope that this helps position our contribution more clearly within the emerging social distancing and related Covid-19 literature.
